# Extract of *Polygala tenuifolia*, *Angelica tenuissima*, and *Dimocarpus longan* improve skin wound healing in streptozotocin- induced diabetic mouse

**DOI:** 10.3389/fphar.2026.1779494

**Published:** 2026-03-03

**Authors:** Hayan Jeong, Hyo-Jin Chong, Yejin Jo, Jungtae Na, Yeon Jae Jang, Su Young Moon, Jangho So, In Ho Jung, Ok Nam Park, Bong-Gun Ju

**Affiliations:** 1 Department of Life Science, Sogang University, Seoul, Republic of Korea; 2 R&D Center, Medi Help Line Co., Seoul, Republic of Korea

**Keywords:** chronic skin wound, diabetic wound healing, growth factor, inflammation, WIN-1001X

## Abstract

**Objective:**

Chronic skin wounds caused by diabetes, peripheral artery disease, pressure ulcers, and venous insufficiency do not fully recover anatomically and functionally. We previously found that topical application of 3% WIN-1001X cream reduces skin inflammation. In this study, we investigated whether 3% WIN-1001X cream alleviates chronic skin wounds exhibiting prolonged and extensive inflammation using streptozotocin-induced diabetic mouse.

**Methods:**

WIN-1001X contained 20% ethanol extracts of three botanical drugs: *Polygala tenuifolia* Willd., *Angelica tenuissima* Nakai, and *Dimocarpus longan* Lour. Streptozotocin-induced diabetic mice were used as a chronic wound model. After making full-thickness excision wounds were made on shaved dorsal skin, 3% WIN-1001X cream was topically applied daily for 12 days. The wound area was measured, and histology was performed to detect granulation tissue and collagen deposition. Quantitative real-time PCR and immunohistochemistry were performed to measure expression of RNA and proteins related to wound healing such as pro-inflammatory cytokines, growth factors, anti-microbial peptides, cell proliferation, keratinocyte differentiation, myofibroblast formation, and macrophage infiltration.

**Results:**

Topical application of 3% WIN-1001X cream suppressed infiltration of neutrophils and monocytes as well as pro-inflammatory cytokine gene activation in diabetic mouse skin. It also promotes M1 to M2 macrophage polarization. Interestingly, 3% WIN-1001X cream activated the gene expression of anti-microbial peptides. Furthermore, It upregulated gene expression of *PDGFβ, HGF, KGF*, and *TGFβ*, resulting in the promotion of cell proliferation, granulation tissue formation, myofibroblast formation, and keratinocyte differentiation.

**Conclusion:**

3% WIN-1001X cream suppressed skin inflammation through decreased cytokine gene expression, immune cell infiltration, and increased macrophage polarization. It also promoted cell proliferation, granulation tissue formation, and myofibroblast transition. Furthermore, 3% WIN-1001X cream promoted keratinocyte re-epithelialization and differentiation as well as increased collagen deposition in chronic skin wounds. Thus, our results suggest that 3% WIN-1001X cream may help alleviate chronic skin wounds.

## 1 Introduction

Skin wounds heal naturally through four stages: hemostasis, inflammation, proliferation, and dermal remodeling (Pastar et al., 2014; Rodrigues et al., 2019; Wilkinson and Hardman, 2020). In hemostasis, platelets bind to the extracellular matrix (ECM) in the vessel wall and form blood clots to prevent hemorrhage. They also secrete various chemokines and growth factors, which are important for the recruitment of immune cells to the wound site and the stimulation of resident cells such as fibroblasts and keratinocytes. Then, signals from damaged tissues and necrotic cells, as well as bacterial components, evoke immune responses by activating immune cells including mast cells, neutrophils, macrophages, and T cells. These cells play critical role in phagocytosis and the production of cytokines and growth factors for cell proliferation, migration, and angiogenesis. In the proliferation phase, proliferated fibroblasts synthesize disorganized collagen, keratinocytes migrate from the wound edges to form epithelial layer, and endothelial cells also migrate from existing blood vessels into the wound site, contributing to granulation tissue formation, re-epithelialization, and neovascularization. In the final remodeling phase, granulation tissues matures into permanent scar. Additionally, type III reticular collagen is replaced with type I fibrillar collagen.

Chronic wounds refer to wounds that do not fully recover anatomically and functionally within 3 months during the wound healing process (Han and Ceilley, 2017; Berthiaume and Hsia, 2022). Chronic wounds are induced by conditions such as diabetes, peripheral artery disease, pressure ulcers, and venous insufficiency. In particular, diabetic wounds exhibit nerve damage, reduced blood flow, and impaired immune responses (Patel et al., 2019; Burgess et al., 2021). Consequently, diabetic skin wounds impede the normal phases of wound healing, including hemostasis, inflammation, proliferation, and tissue remodeling. In the inflammation phase, the M1 macrophage phenotype predominates and plays a key role in the chronic nature of diabetic wounds (Aitcheson et al., 2021; Burgess et al., 2021; Sim et al., 2022). Impairment of the transition process from M1 to M2 macrophages reduces the rate of wound healing, leading to impaired wound closure, impaired angiogenesis, and reduced collagen deposition (Aitcheson et al., 2021; Sim et al., 2022). Although various drugs for chronic skin wounds, including growth factors, have been developed, addressing these wounds remains challenging due to their complicated features of wound healing (Han and Ceilley, 2017; Spampinato et al., 2020).

WIN-1001X is a 20% ethanol extract of *Polygala tenuifolia*, *Angelica tenuissima*, and *Dimocarpus longan* combined in a 1:1:1 ratio (Li et al., 2021). It is also a modified Korean traditional botanical drug formula ‘Chungsimyeolda-tang’ which has been well described in the historic text ‘Dongui Sasang Shinpyun’ (Shim et al., 2008). In this study, we investigated whether WIN-1001X alleviates chronic skin wounds induced in streptozotocin (STZ)-induced diabetic.

## 2 Materials and methods

### 2.1 Preparation of WIN-1001X

WIN-1001X is composed of 20% ethanol extracts of three botanical drugs; *Polygala tenuifolia* Wildenow [Polygalaceae; Polygalae radix], *Angelica tenuissima* Nakai [Apiaceae; Angelicae tenuissimae radix], and *Dimocarpus longan* Loureiro [Sapindaceae; Longan arillus] which were purchased from Booyoung Pharmacy (Seoul, Korea). These three medicinal botanical drugs were mixed in a weight ratio of 1:1:1. The identification and authentication of these botanical drugs were verified by KGC Yebon Co., Ltd. (Cheongju-si, Korea), and specimens were stored in the Medihelpline Research Center plant specimen room (Seoul, Korea). The mixture was reflux-extracted for 3 h with 6 times the weight of the raw material in 20% ethanol (v/v) and then filtered. The residue was subsequently reflux-extracted for another 3 h with 4 times the weight of the raw material in 20% ethanol (v/v) and filtered. The combined filtrates were concentrated under reduced pressure and lyophilized to obtain the final dry extract. The drug-to-extract ratio (DER) is 3:1, meaning 1 g of the final extract (WIN-1001X) corresponds to 3 g of the initial raw botanical drug mixture. The preparation of WIN-1001X was conducted at KGC Yebon Co., Ltd. The analytical results for WIN-1001X are provided in Supplementary Figures 2–6.

### 2.2 UPLC-MS analysis

To confirm chemical compositions and consistent preparation of WIN-1001X from batch to batch, the UPLC-MS analysis was performed using a single quadrupole mass spectrometer (MSD, Agilent 6120, Santa Clara, CA, USA) coupled with Waters ACQUITY ultra performance liquid chromatography (UPLC) system (Waters, Milford, USA). An ACQUITY BEH C18 column (3.0 × 150 mm, 1.7 µm) was used and the mobile phase was composed of solvent A (0.2% acetic acid in H2O) and solvent B (0.2% acetic acid in acetonitrile). The elution gradient proceeded as follows: 0–3 min, 5% B; 3–53 min, 28% B; 53–55 min, 34% B; 55–75 min, 38% B; 75–76 min, 70% B; 76–80 min, 70% B. The flow rate is maintained at 0.5 mL/min and injection volume is 10 µL. The column oven is maintained at a temperature of 30 °C. The mass spectrometry parameters were as follows: positive ion mode; gas temperature, 350 °C; drying gas, 12 L/min; capillary voltage, +4,000 V; m/z range, 200–1,500. The quantitation of each major metabolite was made using a Photodiode Array (PDA) detector set at 320 nm.

### 2.3 Preparation of WIN-1001X cream

The WIN-1001X cream contained 3% (w/w) of WIN-1001X, respectively. Polyethylene glycol 400 was added as a base, and WIN-1001X was dissolved at 72∼78 °C for 20 min. Then, with the addition of other base ingredients and preservatives, the manufacturing process followed oil phase preparation, cooling, filling and packaging processes in accordance with the manufacturing method of ointment in the General Regulations of the Korean Pharmacopoeia. The 3% WIN-1001X cream was formulated with the following composition (w/w): WIN-1001X (3.0%), propylene glycol (8.0%), heavy liquid paraffin (5.0%), cetanol (2.5%), stearyl alcohol (5.0%), stearic acid (5.0%), isopropyl myristate (4.0%), sorbitan monostearate (3.0%), polysorbate 60 (3.0%), methyl parahydroxybenzoate (0.1%), and propyl parahydroxybenzoate (0.05%). The mixture was homogenized to ensure uniform dispersion. The preparation of WIN-1001X cream was conducted by Cires Pharmaceutical Inc. (Hwaseong-si, Korea).

### 2.4 Animal model of chronic skin wound

Adult male C57BL/6J mice (7 weeks old, DBL, Korea) were maintained in a temperature-controlled room (23 °C) at 55% humidity, with a 12-h light-dark cycle. The Committee for Experimental Animal Research at Sogang University approved the animal experiments (IACUCSGU2021_11). Diabetes was induced by intraperitoneal injection of streptozotocin (ALX-380-010-G001, ENZO) for 5 days. Streptozotocin was dissolved in 0.5 M sodium citrate buffer (pH 4.5). Whole blood from the mouse tail vein was measured using a blood glucose monitoring system (Accu-CheK Rerforma, Roche). On day 25, mice with a blood glucose value ≥300 mg/dL, were defined as streptozotocin-induced diabetic mice. On day 28, the mouse was anesthetized with 2,2,2-tribromoethanol and a full-thickness excision wound was made on the shaved dorsal skin using a 6 mm biopsy punch (Kai Industries) (n = 6 mice). WIN-1001X cream was topically applied daily for 12 days. All animal experiments were indeed performed three times independently, with n = 6 mice per group in each iteration. Data analysis was performed in a blinded manner. Samples were coded, and investigators were unaware of the treatment groups during measurement and histological evaluation.

### 2.5 Quantitative PCR

Total RNA was extracted from mouse skin wounds using Tri-RNA Reagent (TR118, Favorgen). First-strand cDNA synthesis was performed with PrimeScript RT master mix (RR036A, Takara). The resulting cDNAs were subjected to real-time PCR using qPCR 2x Premix SYBR (RT500M, Enzynomics) with a QuantStudio 1 Real-Time PCR System (Applied Biosystems). The PCR conditions were 10 min at 95 °C, followed by 40 cycles of 95 °C for 15 s and 64 °C for 40 s. Expression data were calculated from the cycle threshold (Ct) value using the ΔCt method of quantification. *18s rRNA* was used for normalization. Oligonucleotides are listed in Supplementary Table 1.

### 2.6 Histology

Mouse dorsal skin samples were immediately fixed with 10% neutral buffered formalin (0144, Medilab, Korea) and left overnight at 4 °C. The samples were dehydrated, embedded in paraffin, and sectioned at 5 μm. The tissue sections were deparaffinized with xylene twice for 10 min each. Rehydration of sections was serially performed with 100%, 95%, 70%, 50% ethanol, followed by tap water. Tissue sections were stained with hematoxylin and eosin. Mast cells and collagen were stained with toluidine blue and Masson’s trichrome stain, respectively.

### 2.7 Immunohistochemistry

Rehydrated tissue sections were autoclaved in sodium citrate buffer (pH 6.0) for 10 min. After cooling, sections were washed in PBST (0.1% Triton X-100 in PBS) for 5 min and blocked with 5% BSA in 0.1% PBST for 1 h. For immunofluorescence, tissue sections were incubated with anti-arginase1 (66129-1-Ig, Proteintech), anti-PCNA (ab15497, Abcam), and anti-cytokeratin 17 (sc-393002, Santa Cruz Biotechnology) antibodies overnight at 4 ℃,respectively . A secondary antibody conjugated to Cy5 (ab6563, ab6564, Abcam) was used. For DAB staining immunohistochemistry, tissue sections were incubated with anti-MPO (PA5-16672, Invitrogen), anti-iNOS (BD 610329, BD Bioscience), anti-VEGF (sc-152, Santa Cruz Biotechnology), and anti-αSMA (sc-32251, Santa Cruz Biotechnology) antibodies. HRP/DAB (ABC) detection IHC kit (ab64264, Abcam) was used. For DAB immunostaining, tissue sections were incubated with anti-MPO (PA5-16672, Invitrogen) and anti-iNOS (BD 610329, BD bioscience), anti-VEGF (sc-152, Santa Cruz Biotechnology), anti-αSMA (sc-32251, Santa Cruz Biotechnology) antibodies overnight at 4 ℃ . Then, the HRP/DAB (ABC) detection IHC kit (ab64264, Abcam) was used according to the manufacturer’s instructions. Expression was quantified using ImageJ software (NIH, Bethesda, MD, USA).

### 2.8 Statistical analysis

All animal experiments were indeed performed independently three times, with n = 6 mice per group in each experiment. All quantitative data were presented as the mean ± standard error of the mean (S.E.M.) from three independent experiments. Statistical differences among multiple groups were analyzed by one-way or two-way analysis of variance (ANOVA) followed by Dunnett’s post hoc test. Statistical significance was defined as *P ≤ 0.05, **P ≤ 0.01, and ***P ≤ 0.005. All p-values indicated in the figures are listed in Supplementary Table 2.

## 3 Results

### 3.1 3% WIN-1001X cream accelerates chronic skin wound healing

**FIGURE 1:**
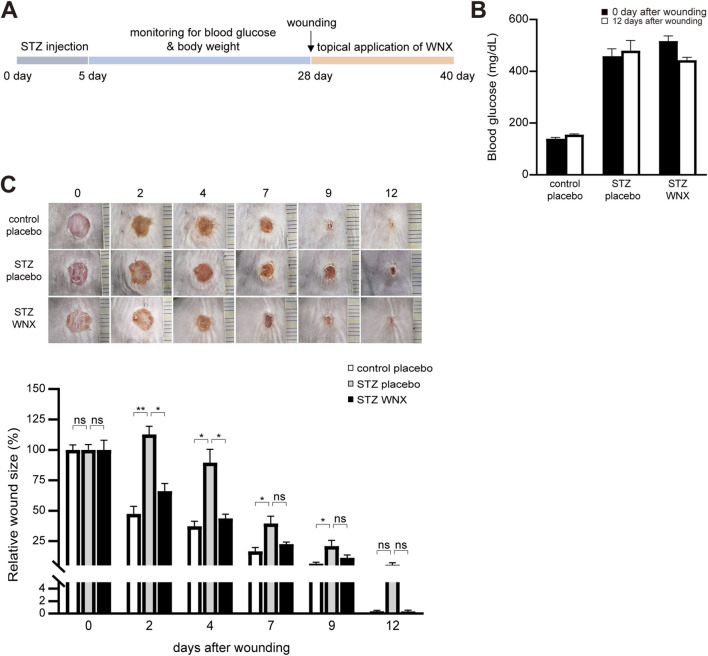
3% WIN-1001X cream accelerates chronic skin wound healing. **(A)** Experimental scheme. After diabetes was induced by intraperitoneal injection of streptozotocin (STZ), full-thickness excisional wounds were created in the shaved dorsal skin. Three percent of WIN-1001X cream (WNX) was topically applied every day for 12 days after wounding (see Materials and Methods). **(B)** Fasting blood glucose levels was measured at 0 and 12 days after wounding. Mice with fasting blood glucose levels exceeding 300 mg/dL were included for this study. **(C)** Topical application of 3% WIN-1001X cream accelerates chronic skin wound healing in diabetic mice (n = 6/per group). Normal wounds topically applied with placebo cream were used as controls. The wound was photographed and representative images are shown. The wound area was quantified using the ImageJ software. All data represent mean ± S.E.M. Statistical significance was indicated as *P ≤ 0.05, **P ≤ 0.01, and ***P ≤ 0.005.

We previously found that topical application of WIN-1001X reduces skin inflammation (manuscript in preparation). Thus, we further tested whether WIN-1001X cream alleviates chronic skin wounds that exhibit prolonged and extensive inflammation (Rosique et al., 2015; Zhao et al., 2016; Burgess et al., 2021). We used streptozotocin (STZ)-induced diabetic mice with fasting blood glucose levels exceeding 300 mg/dL as a chronic skin wound model (Figures 1A,B). For topical application, different percentages of WIN-1001X cream (WNX) were prepared and tested the efficacy of WIN-1001X cream in chronic skin wound healing (Supplementary Figure 1). We finally selected 3% WIN-1001X cream for further study. Although diabetic mouse treated with placebo cream (STZ placebo) showed delayed skin wound healing compared with normal skin wounds treated with placebo cream (control placebo), topical application of 3% WIN-1001X cream (STZ WNX) accelerated skin wound healing in diabetic mice (Figure 1C).

### 3.2 3% WIN-1001X cream suppresses infiltration of immune cells in chronic skin wounds

**FIGURE 2:**
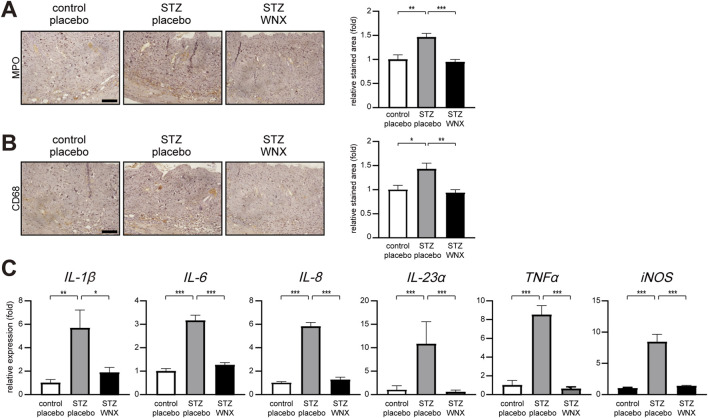
3% WIN-1001X cream suppresses skin inflammation in chronic skin wounds. **(A,B)** Topical application of 3% WIN-1001X cream (WNX) suppresses infiltration of neutrophils and macrophages in chronic skin wounds (n = 6/group). At 2 days after topical application of 3% WIN-1001X cream, skin tissues were harvested. Skin sections were immunostained with anti-MPO (myeloperoxidase) and anti-CD68 antibodies for the detection of neutrophils and macrophages, respectively. Normal wounds topically applied with placebo cream were used as controls. Expression was quantified using the ImageJ software. Representative images are shown. Scale bar, 50 μm. **(C)** Topical application of 3% WIN-1001X cream suppresses activation of proinflammatory cytokines and *iNOS* genes in chronic skin wounds (n = 6/per group). At 2 days after topical application of 3% WIN-1001X cream, skin tissues were harvested. Transcripts of *IL-1β, IL-6, IL-8, IL-23α, TNFα, iNOS,* and *18S rRNA* were quantified using real-time RT-PCR (n = 6). All data represent mean ± S.E.M. Statistical significance was indicated as *P ≤ 0.05, **P ≤ 0.01, and ***P ≤ 0.005.

Given the extensive and prologned inflammatory phase in diabetic skin wounds (Rosique et al., 2015; Zhao et al., 2016; Burgess et al., 2021), we next examined the infiltration of immune cells into the skin wound. The infiltration of MPO (myeloperoxidase)-positive neutrophils was increased in diabetic skin wounds compared with normal skin wounds at 2 days after wounding. However, topical application of 3% WIN-1001X cream reduced the infiltration of neutrophils in diabetic skin wounds (Figure 2A). The infiltration of CD68-positive monocytes and macrophages in diabetic skin wounds treated with 3% WIN-1001X cream was also reduced compared with diabetic skin wounds treated with placebo cream at 2 days after wounding (Figure 2B). Consistently, the topical application of 3% WIN-1001X cream suppressed the upregulation of the gene expression of pro-inflammatory cytokines, including *IL-1β, IL-6, IL-8, IL-23α,* and *TNFα* as well as *iNOS* in diabetic skin wounds at 2 days after wounding (Figure 2C).

### 3.3 3% WIN-1001X cream upregulates gene expression of anti-microbial peptides in chronic skin wounds

**FIGURE 3:**
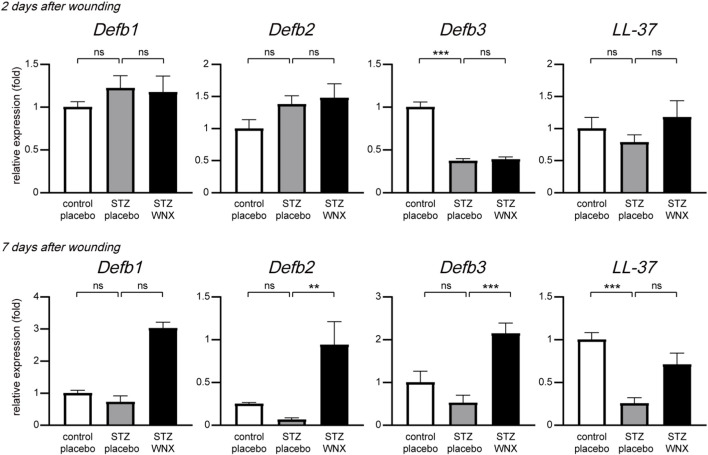
3% WIN-1001X cream upregulates gene expression of antimicrobial peptides in chronic skin wounds. Topical application of 3% WIN-1001X cream upregulates the gene expression of antimicrobial peptides in chronic skin wounds (n = 6/group). At 2 and 7 days after topical application of 3% WIN-1001X cream, skin tissues were harvested. Transcripts of *Defb1, Defb2, Defb3, LL-37,* and *18S rRNA* were quantified using real-time RT-PCR (n = 6). All data represent mean ± S.E.M. Statistical significance was indicated as *P ≤ 0.05, **P ≤ 0.01, and ***P ≤ 0.005.

Microbial infection delays wound healing and often results in serious outcomes such as surgical debridement (Frykberg and Banks, 2015; Negut et al., 2018). WIN-1001X has no direct antimicrobial activity against bacteria including *Staphylococcus aureus* and *Pseudomonas aeruginosa* (data not shown). The skin has antimicrobial activity as part of the early stage of the immune defense system through the expression of antimicrobial peptides (Yamasaki and Gallo, 2008; Rademacher et al., 2021). We thus tested whether 3% WIN-1001X cream regulates the gene expression of antimicrobial peptides in diabetic skin. At 2 days after wounding, the topical application of 3% WIN-1001X cream had no effect on the expression of *Defb1, Defb2, Defb3,* and *LL-37* genes in diabetic skin wounds. However, upregulation of *Defb2, Defb3*, and *LL-37* gene expression was observed in 3% WIN-1001X cream-treated diabetic skin wounds at 7 days after wounding (Figure 3).

### 3.4 3% WIN-1001X cream promotes M1 to M2 macrophage polarization in chronic skin wounds

**FIGURE 4:**
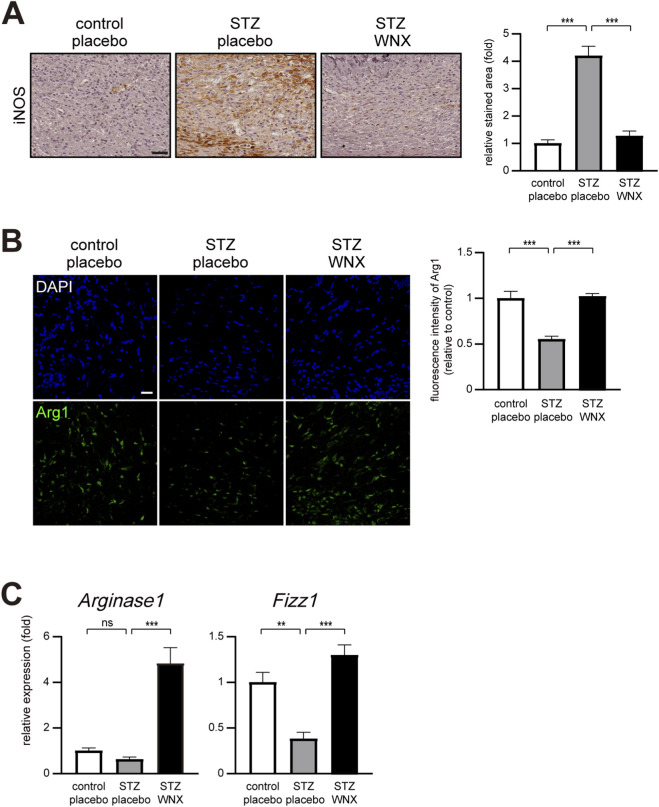
3% WIN-1001X cream promotes M1 to M2 macrophage polarization in chronic skin wounds. **(A)** Topical application of 3% WIN-1001X cream suppresses excessive infiltration of M1 macrophage in chronic skin wounds (n = 6/per group). At 2 days after topical application of 3% WIN-1001X cream, skin tissues were harvested. Skin sections were immunostained with anti-iNOS antibody for the detection of M1 macrophages. Normal wounds applied with placebo cream were used as controls. Expression was quantified using the ImageJ software. Representative images are shown. Scale bar, 50 μm. **(B,C)** Topical application of 3% WIN-1001X cream promotes infiltration of M2 macrophages in chronic skin wounds (n = 6/group). At 7 days after topical application of 3% WIN-1001X cream, skin tissues were harvested. Skin sections were immunostained with anti-Arg1 (Arginase 1) antibody for the detection of M2 macrophages. Normal wounds applied with placebo cream were used as controls. Expression was quantified using the ImageJ software. Representative images are shown. Scale bar, 50 μm. Transcripts of *Arg1, Fizz1*, and *18S rRNA* were quantified using real-time RT-PCR (n = 6). All data represent mean ± S.E.M. Significance values were *P ≤ 0.05, **P ≤ 0.01, and ***P ≤ 0.005.

Classically activated M1 macrophages, which promote inflammation, could be polarized into alternatively activated M2 macrophages for successful wound repair (Ferrante and Leibovich, 2012; Louiselle et al., 2021). The prolonged existence of M1 macrophages is one of the characteristics of chronic wound healing. Thus, we examined the existence of M1 and M2 macrophages at 7 days after wounding by immunostaining with anti-iNOS (inducible NOS) and anti-Arg1 (Arginase 1) antibodies, respectively. The increased level of iNOS-positive M1 macrophages was maintained in diabetic skin wounds compared with normal skin wounds at 7 days after wounding (Figure 4A). However, 3% WIN-1001X cream treatment restored the normal level of M1 macrophages in diabetic skin wounds (Figure 4A). Additionally, 3% WIN-1001X cream maintained the normal level of Arg1-positive M2 macrophages in diabetic skin wounds at 7 days after wounding (Figure 4B). Consistent with immunohistochemical results, 3% WIN-1001X cream induces gene expression of markers of M2 macrophages such as *Arg1* and *Fizzl* (Figure 4C).

### 3.5 3% WIN-1001X cream promotes cell growth in chronic skin wounds

**FIGURE 5:**
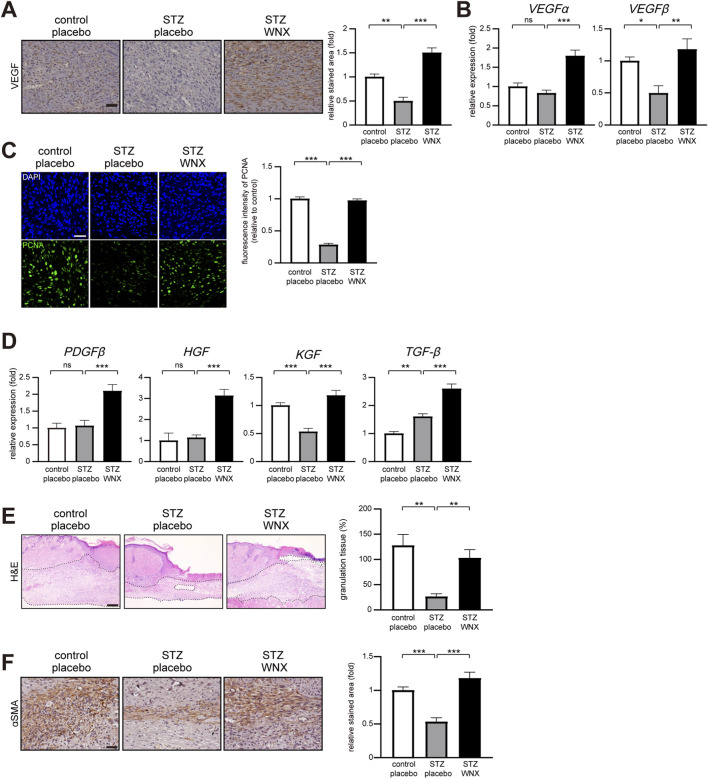
3% WIN-1001X cream promotes cell proliferation in chronic skin wounds. **(A,B)** Topical application of 3% WIN-1001X cream upregulates VEGF expression in chronic skin wounds (n = 6/per group). At 7 days after topical application of 3% WIN-1001X cream, skin tissues were harvested. Skin tissue sections were immunostained with anti-VEGF antibody. Expression was quantified using the ImageJ software. Representative images are shown. Transcripts of *VEGFα, VEGFβ,* and *18S rRNA* were quantified using real-time RT-PCR (n = 6). **(C)** Topical application of 3% WIN-1001X cream increases cell proliferation in chronic skin wounds (n = 6/per group). At 7 days after topical application of 3% WIN-1001X cream, skin tissues were harvested. Skin sections were immunostained with anti-PCNA antibody. Normal wounds applied with placebo cream were used as controls. Expression was quantified using the ImageJ software. Representative images are shown. Scale bar, 50 μm. **(D)** Topical application of 3% WIN-1001X cream upregulates the gene expression of growth factors in chronic skin wounds (n = 6/per group). At 7 days after topical application of 3% WIN-1001X cream, skin tissues were harvested. Transcripts of *PDGFβ, HGF, KGF, TGFβ,* and *18S rRNA* were quantified using real-time RT-PCR (n = 6). **(E)** Topical application of 3% WIN-1001X cream promotes the formation of granulation tissues in chronic skin wounds (n = 6/per group). At 7 days after topical application of 3% WIN-1001X cream, skin tissues were harvested. Skin sections were stained with hematoxylin and eosin. Normal wounds applied with placebo cream were used as controls. The area of granulation tissues was quantified using the ImageJ software. Representative images are shown. Scale bar, 100 μm. **(F)** Topical application of 3% WIN-1001X cream promotes myofibroblast differentiation in chronic skin wounds (n = 6/per group). At 7 days after topical application of 3% WIN-1001X cream, skin tissues were harvested. Skin sections were immunostained with anti-αSMA (smooth muscle actin α) antibody. Expression was quantified using the ImageJ software. Representative images are shown. Scale bar, 50 μm. All data represent mean ± S.E.M. Statistical significance was indicated as *P ≤ 0.05, **P ≤ 0.01, and ***P ≤ 0.005.

VEGF (vascular endothelial growth factor) plays a role in angiogenesis in wound healing through endothelial cell proliferation, migration, differentiation, and survival (Bao et al., 2009; Johnson and Wilgus, 2014). We found that topical application of 3% WIN-1001X upregulated VEGF expression in diabetic skin wounds compared with the placebo control at 7 days after wounding (Figures 5A,B). Additionally, the transition from inflammation to proliferation is also a critical process for successful wound healing (Landén et al., 2016). We examined the cell proliferation of keratinocytes and fibroblasts by immunostaining using anti-PCNA (proliferating cell nuclear antigen) antibody. Topical application of 3% WIN-1001X promoted keratinocytes and fibroblasts proliferation in diabetic skin wounds compared with placebo-treated diabetic skin wounds at 7 days after wounding (Figure 5C). Moreover, 3% WIN-1001X cream activated the gene expression of growth factors including *PDGFβ, HGF, KGF and TGFβ*, which are responsible for cell proliferation in diabetic skin wounds compared with diabetic skin wounds treated with placebo cream (Figure 5D). An increase in granulation tissue formation was observed in 3% WIN-1001X cream-treated diabetic skin wounds compared with placebo cream-treated diabetic skin wounds at 7 days after wounding (Figure 5E). Additionally, we observed that 3% WIN-1001X cream promoted myofibroblast formation in granulation tissue (Figure 5F).

### 3.6 3% WIN-1001X cream promotes re-epithelialization and collagen deposition in chronic skin wounds

**FIGURE 6:**
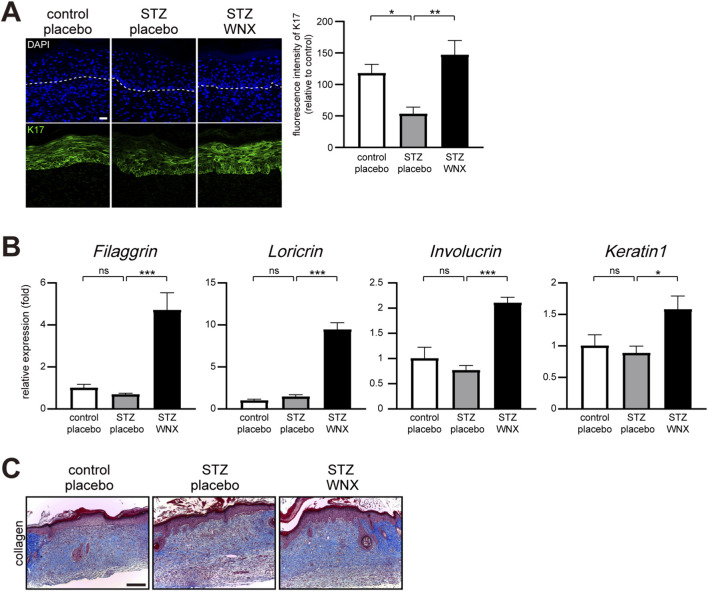
3% WIN-1001X cream promotes reepithelialization and tissue remodeling in chronic skin wounds. **(A)** Topical application of 3% WIN-1001X cream promotes reepithelialization in chronic skin wounds (n = 6/per group). At 7 days after topical application of 3% WIN-1001X cream, skin tissues were harvested. Skin sections were immunostained with anti-K17 antibody. Expression was quantified using the ImageJ software. Representative images are shown. Scale bar, 50 μm. **(B)** Topical application of 3% WIN-1001X cream promotes keratinocyte differentiation in chronic skin wounds (n = 6/per group). At 7 days after topical application of 3% WIN-1001X cream, skin tissues were harvested. Transcripts of *Filaggrin, Loricrin, Involucrin, Keratin 1*, and *18S rRNA* were quantified using real-time RT-PCR (n = 6). **(C)** Topical application of 3% WIN-1001X cream promotes collagen synthesis in chronic skin wounds (n = 6/per group). At 12 days after topical application of 3% WIN-1001X cream, skin tissues were harvested. Skin sections were stained with Masson’s trichrome. Expression was quantified using the ImageJ software. Representative images are shown. Scale bar, 100 μm. All data represent mean ± S.E.M. Statistical significance was indicated as *P ≤ 0.05, **P ≤ 0.01, and ***P ≤ 0.005.

Immunostaining with an anti-K17 (keratin 17) antibody showed that topical application of 3% WIN-1001X cream promoted re-epithelialization as a result of epithelial keratinocyte migration over the wound bed in chronic skin wounds at 7 days after wounding (Figure 6A). We further examined the expression of genes related to keratinocyte differentiation. Topical application of 3% WIN-1001X cream upregulated *Filaggrin, Loricrin, Involucrin, and Keratin 1* gene expression in chronic skin wounds at 7 days after wounding (Figure 6B). We also found that topical application of 3% WIN-1001X cream promoted collagen deposition, as observed in tissue sections stained with Masson’s trichrome (Figure 6C).

## 4 Discussion

WIN-1001X is a 20% ethanol extract of *Polygala tenuifolia*, *Angelica tenuissima*, and *Dimocarpus longan* combined in a 1:1:1 ratio (Li et al., 2021). We recently found that topical application of WIN-1001X reduces skin inflammation (manuscript in preparation). In this study, we further investigated whether WIN-1001X alleviates chronic skin wounds that exhibit prolonged and extensive inflammation as well as other abnormal healing processes (Rosique et al., 2015; Zhao et al., 2016; Burgess et al., 2021). In fact, a persistent inflammation state disrupts normal skin wound healing progress and increases scar formation (Eming et al., 2007; Wilgus, 2020). Thus, the transition from the inflammation stage to the proliferation stage may be a key therapeutic intervention for chronic skin wounds.

Our results demonstrated that 3% WIN-1001X cream suppressed extensive inflammation by suppressing the of infiltration of neutrophils and monocytes and cytokine gene expression such as including *IL-1β, IL-6, IL-8, IL-23α*, and *TNFα* compared with placebo- treated chronic wounds. In addition, we found that 3% WIN-1001X cream promoted pro-inflammatory M1 to anti-inflammatory M2 macrophage polarization, which is required for successful wound repair (Ferrante and Leibovich, 2012; Louiselle et al., 2021). Additionally, oral and topical toxicity tests were conducted for WIN-1001X, and the data confirmed its safety (Supplementary Table 3). Indeed, extracts of each plant have been reported to have anti-inflammatory activity. The root extract of *Polygala tenuifolia* suppressed lipopolysaccharide (LPS)-stimulated upregulation of *iNOS, COX-2, TNFα*, and *IL-1β* in BV2 microglial cells via inhibition of IκB-α degradation (Cheong et al., 2011). The extract of *Angelica tenuissima* exhibited anti-inflammatory effects by suppressing of calcium release, as well as p38MAPK and STAT3 phosphorylation in LPS-treated RAW264.7 macrophages (Kim et al., 2022). In addition, the extracts of *Dimocarpus longan* inhibited LPS-induced degradation of IκBα and the activation of NF-κB, AP-1, Akt, and MAP kinases in RAW264.7 macrophages (Kunworarath et al., 2016).

We also have identified active metabolites of WIN-1001X using UPLC-MS analysis (Supplementary Figure 7). Interestingly, previous have demonstrated that some of the active metabolites of *Polygala tenuifolia*, *Angelica tenuissima*, and *Dimocarpus longan* have anti-inflammatory effects. For instance, tenuigenin, tenuifoliside A, tenuifolin, and senegenin isolated from *Polygala tenuifolia* suppressed inflammation by various mechanisms such as the inhibition of NF-κB and NLRP3 inflammasome or by activation of NRF2-HO1 signaling (Yuan et al., 2012; Kim et al., 2013; Lv et al., 2016; Wang et al., 2016; Lu et al., 2017; Wang et al., 2017; Li et al., 2017; Chen and Jia, 2020). In addition, the 3-*O*-(3,4,5-trimethoxy-cinnamoyl), 6′-*O*-(*p*-methoxybenzoyl) sucrose ester from *Polygala tenuifolia* inhibited expression of *the iNOS, COX-2, TNF-α, IL-1β,* and *IL-6* (Son et al., 2022). Onjisaponin B of WIN-1001X also reduces the level of *TNF-α, IL-1β,* and *IL-6* in LPS-treated PC12 cells (Li et al., 2018; Li et al., 2021). Decursin and (Z)-ligustilide from *Angelica tenuissima* extract also have anti-inflammatory effects in skin wound healing. Decursin decreased LPS-induced oxidative stress and inflammation through suppression of activation of the NF-κB pathway (Zhu and Dong, 2023). (Z)-ligustilide also suppressed activation of the NF-κB pathway via inhibition of gene expression and signaling (Wang et al., 2010; Chung et al., 2012; Choi et al., 2018).

Topical application of 3% WIN-1001X cream further promoted cell proliferation, granulation tissue formation, myofibroblast formation, and collagen deposition in skin wound healing. Although the exact active metabolite of WIN-1001X responsible for these wound healing process is not currently known, polygalaxanthone III, a xanthone glycoside of *Polygala tenuifolia* has been reported to promote skin wound healing induced by yeast infection through the STAT3 pathway (Tsujimoto et al., 2019; Yang et al., 2022). Additionally, decursin improves keratinocyte wound healing by upregulating the expression of genes encoding extracellular matrix remodeling proteins and growth factors (Han et al., 2018).

In conclusion, we studied the efficacy of 3% WIN-1001X cream in chronic skin wounds using streptozotocin-induced diabetic mice. Specifically, 3% WIN-1001X cream suppressed skin inflammation by decreasing cytokine gene expression and immune cell infiltration, and by increasing macrophage polarization. It also promoted cell proliferation, granulation tissue formation, and myofibroblast transition. Furthermore, 3% WIN-1001X cream promoted keratinocyte re-epithelialization and differentiation as well as increased collagen deposition in chronic skin wounds. Thus, our results suggest that 3% WIN-1001X cream may help alleviate chronic skin wounds, for which currently no effective therapeutics exist.

## 5 Conclusion

3% WIN-1001X cream suppressed skin inflammation by decreasing cytokine gene expression and immune cell infiltration, and by increasing macrophage polarization. It also promoted cell proliferation, granulation tissue formation, and myofibroblast transition. Furthermore, 3% WIN-1001X cream promoted keratinocyte re-epithelialization and differentiation as well as increasing collagen deposition in chronic skin wounds. Thus, our results suggest that 3% WIN-1001X cream may help alleviate chronic skin wounds.

## Data Availability

The original contributions presented in the study are included in the article/Supplementary Material, further inquiries can be directed to the corresponding author.

## References

[B1] Aitcheson S. M. Frentiu F. D. Hurn S. E. Edwards K. Murray R. Z. (2021). Skin wound healing: normal macrophage function and macrophage dysfunction in diabetic wounds. Molecules 26 (16), 4917. 10.3390/molecules26164917 34443506 PMC8398285

[B2] Bao P. Kodra A. Tomic-Canic M. Golinko M. S. Ehrlich H. P. Brem H. (2009). The role of vascular endothelial growth factor in wound healing. J. Surg. Res. 153 (2), 347–358. 10.1016/j.jss.2008.04.023 19027922 PMC2728016

[B3] Berthiaume F. Hsia H. C. (2022). Regenerative approaches for chronic wounds. Annu. Rev. Biomed. Eng. 24, 61–83. 10.1146/annurev-bioeng-010220-113008 35226819

[B4] Burgess J. L. Wyant W. A. Abdo Abujamra B. Kirsner R. S. Jozic I. (2021). Diabetic wound-healing science. Med. Kaunas. 57 (10), 1072. 10.3390/medicina57101072 34684109 PMC8539411

[B5] Chen S. Jia J. (2020). Tenuifolin attenuates Amyloid-β42-Induced neuroinflammation in microglia through the NF-κB signaling pathway. J. Alzheimers Dis. 76 (1), 195–205. 10.3233/JAD-200077 32444542

[B6] Cheong M. Lee S. Yoo H. Jeong J. Kim G. Kim W. (2011). Anti-inflammatory effects of Polygala tenuifolia root through inhibition of NF-κB activation in lipopolysaccharide-induced BV2 microglial cells. J. Ethnopharmacol. 137 (3), 1402–1408. 10.1016/j.jep.2011.08.008 21856398

[B7] Choi E. S. Yoon J. J. Han B. H. Jeong D. H. Lee Y. J. Kang D. G. (2018). Ligustilide attenuates vascular inflammation and activates Nrf2/HO-1 induction and, NO synthesis in HUVECs. Phytomedicine 38, 12–23. 10.1016/j.phymed.2017.09.022 29425644

[B8] Chung J. W. Choi R. J. Seo E. K. Nam J. W. Dong M. S. Shin E. M. (2012). Anti-inflammatory effects of (Z)-ligustilide through suppression of mitogen-activated protein kinases and nuclear factor-κB activation pathways. Arch. Pharm. Res. 35 (4), 723–732. 10.1007/s12272-012-0417-z 22553066

[B9] Eming S. A. Krieg T. Davidson J. M. (2007). Inflammation in wound repair: molecular and cellular mechanisms. J. Invest Dermatol 127 (3), 514–525. 10.1038/sj.jid.5700701 17299434

[B10] Ferrante C. J. Leibovich S. J. (2012). Regulation of macrophage polarization and wound healing. Adv. Wound Care New Rochelle. 1 (1), 10–16. 10.1089/wound.2011.0307 24527272 PMC3623587

[B11] Frykberg R. G. Banks J. (2015). Challenges in the treatment of chronic wounds. Adv. Wound Care New Rochelle. 4 (9), 560–582. 10.1089/wound.2015.0635 26339534 PMC4528992

[B12] Han G. Ceilley R. (2017). Chronic wound healing: a review of current management and treatments. Adv. Ther. 34 (3), 599–610. 10.1007/s12325-017-0478-y 28108895 PMC5350204

[B13] Han J. Jin W. Ho N. A. Hong J. Kim Y. J. Shin Y. (2018). Decursin and decursinol angelate improve wound healing by upregulating transcription of genes encoding extracellular matrix remodeling proteins, inflammatory cytokines, and growth factors in human keratinocytes. Biochem. Biophys. Res. Commun. 499 (4), 979–984. 10.1016/j.bbrc.2018.04.031 29626469

[B14] Johnson K. E. Wilgus T. A. (2014). Vascular endothelial growth factor and angiogenesis in the regulation of cutaneous wound repair. Adv. Wound Care (New Rochelle) 3 (10), 647–661. 10.1089/wound.2013.0517 25302139 PMC4183920

[B15] Kim K. S. Lee D. S. Bae G. S. Park S. J. Kang D. G. Lee H. S. (2013). The inhibition of JNK MAPK and NF-κB signaling by tenuifoliside A isolated from Polygala tenuifolia in lipopolysaccharide-induced macrophages is associated with its anti-inflammatory effect. Eur. J. Pharmacol. 721 (1-3), 267–276. 10.1016/j.ejphar.2013.09.026 24076326

[B16] Kim T. Kim D. Park W. (2022). Conioselinum tenuissimum root extract modulates macrophage activation *via* the Calcium–STAT3 pathway. Processes 10 (11), 2238. 10.3390/pr10112238

[B17] Kunworarath N. Rangkadilok N. Suriyo T. Thiantanawat A. Satayavivad J. Longan J. (2016). Dimocarpus longan lour. inhibits lipopolysaccharide-stimulated nitric oxide production in macrophages by suppressing NF-κB and AP-1 signaling pathways. Ethnopharmacol 179, 156–161. 10.1016/j.jep.2015.12.044 26721218

[B18] Landén N. X. Li D. Ståhle M. (2016). Transition from inflammation to proliferation: a critical step during wound healing. Cell Mol. Life Sci. 73 (20), 3861–3885. 10.1007/s00018-016-2268-0 27180275 PMC5021733

[B19] Li H. Lin S. Qin T. Li H. Ma Z. Ma S. (2017). Senegenin exerts anti-depression effect in mice induced by chronic un-predictable mild stress *via* inhibition of NF-κB regulating NLRP3 signal pathway. Int. Immunopharmacol. 53, 24–32. 10.1016/j.intimp.2017.10.001 29031144

[B20] Li X. Sun Y. Wei Y. Zhou L. Liu L. Yin P. (2018). Onjisaponin B (OB) is neuroprotective during cognitive loss through immune-mediated and SIRT1 pathways. Curr. Neurovasc Res. 15 (2), 94–102. 10.2174/1567202615666180528071520 29804532

[B21] Li H. Kim J. Tran H. N. K. Lee C. H. Hur J. Kim M. C. (2021). Extract of Polygala tenuifolia, Angelica tenuissima, and Dimocarpus longan reduces behavioral defect and enhances autophagy in experimental models of parkinson's disease. Neuromolecular Med. 23 (3), 428–443. 10.1007/s12017-020-08643-x 33432492

[B22] Louiselle A. E. Niemiec S. M. Zgheib C. Liechty K. W. (2021). Macrophage polarization and diabetic wound healing. Transl. Res. 236, 109–116. 10.1016/j.trsl.2021.05.006 34089902

[B23] Lu L. Li X. Xu P. Zheng Y. Wang X. (2017). Tenuigenin down-regulates the release of nitric oxide, matrix metalloproteinase-9 and cytokines from lipopolysaccharide-stimulated microglia. Neurosci. Lett. 650, 82–88. 10.1016/j.neulet.2017.04.001 28392358

[B24] Lv H. Ren W. Zheng Y. Wang L. Lu G. Yi P. (2016). Tenuigenin exhibits anti-inflammatory activity *via* inhibiting MAPK and NF-κB and inducing Nrf2/HO-1 signaling in macrophages. Food Funct. 7 (1), 355–363. 10.1039/c5fo00807g 26499342

[B25] Negut I. Grumezescu V. Grumezescu A. M. (2018). Treatment strategies for infected wounds. Molecules 23 (9), 2392. 10.3390/molecules23092392 30231567 PMC6225154

[B26] Pastar I. Stojadinovic O. Yin N. C. Ramirez H. Nusbaum A. G. Sawaya A. (2014). Epithelialization in wound healing: a comprehensive review. Adv. Wound Care (New Rochelle) 3 (7), 445–464. 10.1089/wound.2013.0473 25032064 PMC4086220

[B27] Patel S. Srivastava S. Singh M. R. Singh D. (2019). Mechanistic insight into diabetic wounds: pathogenesis, molecular targets and treatment strategies to pace wound healing. Biomed. Pharmacother. 112, 108615. 10.1016/j.biopha.2019.108615 30784919

[B28] Rademacher F. Gläser R. Harder J. (2021). Antimicrobial peptides and proteins: interaction with the skin microbiota. Exp. Dermatol 30 (10), 1496–1508. 10.1111/exd.14433 34310774

[B29] Rodrigues M. Kosaric N. Bonham C. A. Gurtner G. C. (2019). Wound healing: a cellular perspective. Physiol. Rev. 99 (1), 665–706. 10.1152/physrev.00067.2017 30475656 PMC6442927

[B30] Rosique R. G. Rosique M. J. Farina Junior J. A. (2015). Curbing inflammation in skin wound healing: a review. Int. J. Inflam. 2015, 316235. 10.1155/2015/316235 26356299 PMC4556061

[B31] Shim E. B. Lee S. Kim J. Y. Earm Y. E. (2008). Physiome and sasang constitutional medicine. J. Physiol. Sci. 58 (7), 433–440. 10.2170/physiolsci.RV004208 18928639

[B32] Sim S. L. Kumari S. Kaur S. Khosrotehrani K. (2022). Macrophages in skin wounds: functions and therapeutic potential. Biomolecules 12 (11), 1659. 10.3390/biom12111659 36359009 PMC9687369

[B33] Son S. R. Yoon Y. S. Hong J. P. Kim J. M. Lee K. T. Jang D. S. (2022). Chemical constituents of the roots of Polygala tenuifolia and their anti-inflammatory effects. Plants (Basel) 11 (23), 3307. 10.3390/plants11233307 36501346 PMC9738712

[B34] Spampinato S. F. Caruso G. I. De Pasquale R. Sortino M. A. Merlo S. (2020). The treatment of impaired wound healing in diabetes: looking among old drugs. Pharm. (Basel). 13 (4), 60. 10.3390/ph13040060 32244718 PMC7243111

[B35] Tsujimoto T. Nishihara M. Osumi Y. Hakamatsuka T. Goda Y. Uchiyama N. (2019). Structural analysis of polygalaxanthones, C-Glucosyl xanthones of Polygala tenuifolia roots. Chem. Pharm. Bull. (Tokyo) 67 (11), 1242–1247. 10.1248/cpb.c19-00608 31685751

[B36] Wang J. Du J. R. Wang Y. Kuang X. Wang C. Y. (2010). Z-ligustilide attenuates lipopolysaccharide-induced proinflammatory response *via* inhibiting NF-kappaB pathway in primary rat microglia. Acta Pharmacol. Sin. 31 (7), 791–797. 10.1038/aps.2010.71 20581853 PMC4007734

[B37] Wang C. Zeng L. Zhang T. Liu J. Wang W. (2016). Tenuigenin prevents IL-1β-induced inflammation in human osteoarthritis chondrocytes by suppressing PI3K/AKT/NF-κB signaling pathway. Inflammation 39 (2), 807–812. 10.1007/s10753-016-0309-3 26846886

[B38] Wang X. Li M. Cao Y. Wang J. Zhang H. Zhou X. (2017). Tenuigenin inhibits LPS-Induced inflammatory responses in microglia *via* activating the Nrf2-mediated HO-1 signaling pathway. Eur. J. Pharmacol. 809, 196–202. 10.1016/j.ejphar.2017.05.004 28478071

[B39] Wilgus T. A. (2020). Inflammation as an orchestrator of cutaneous scar formation: a review of the literature. Plast. Aesthet. Res. 7, 54. 10.20517/2347-9264.2020.150 33123623 PMC7592345

[B40] Wilkinson H. N. Hardman M. J. (2020). Wound healing: cellular mechanisms and pathological outcomes. Open Biol. 10 (9), 200223. 10.1098/rsob.200223 32993416 PMC7536089

[B41] Yamasaki K. Gallo R. L. (2008). Antimicrobial peptides in human skin disease. Eur. J. Dermatol 18 (1), 11–21. 10.1684/ejd.2008.0304 18086583 PMC2664254

[B42] Yang X. Xiong B. Yuan Z. Liao H. Liu X. Wu Y. (2022). Polygalaxanthone III, an active ingredient in Polygala japonica houtt., repaired malassezia-stimulated skin injury *via* STAT3 phosphorylated activation. Molecules 27 (21), 7520. 10.3390/molecules27217520 36364345 PMC9655589

[B43] Yuan H. L. Li B. Xu J. Wang Y. He Y. Zheng Y. (2012). Tenuigenin protects dopaminergic neurons from inflammation-mediated damage induced by the lipopolysaccharide. CNS Neurosci. Ther. 18 (7), 584–590. 10.1111/j.1755-5949.2012.00347.x 22759267 PMC6493587

[B44] Zhao R. Liang H. Clarke E. Jackson C. Xue M. (2016). Inflammation in chronic wounds. Int. J. Mol. Sci. 17 (12), 2085. 10.3390/ijms17122085 27973441 PMC5187885

[B45] Zhu J. Dong X. (2023). Decursin alleviates LPS-induced lung epithelial cell injury by inhibiting NF-κB pathway activation. Allergol. Immunopathol. Madr. 51 (1), 37–43. 10.15586/aei.v51i1.689 36617820

