# Supplementary material for: Extract of Polygala tenuifolia, Angelica tenuissima, and Dimocarpus longan improve skin wound healing in streptozotocin- induced diabetic mouse
Source: Front Pharmacol. 2026 Mar 3;17:1779494. doi: 10.3389/fphar.2026.1779494 (PMC13040356; doi:10.3389/fphar.2026.1779494)

**Supplementary Figure 1**


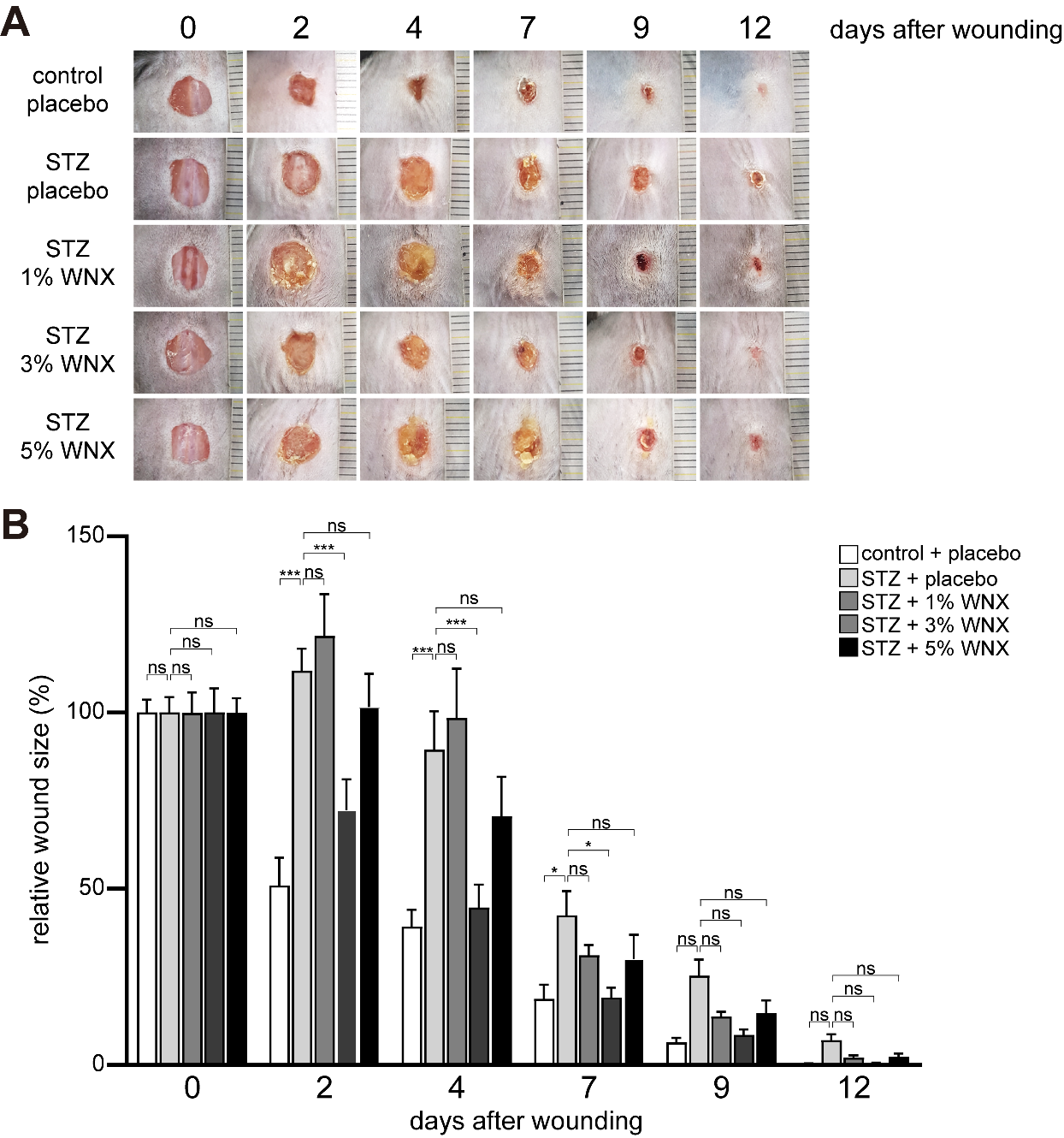


**Supplementary Figure 1. Topical application of 3% WIN-1001X cream accelerates chronic skin wound healing compared to 1% and 5% WIN-1001X creams.** (A, B) After diabetes was induced by an intraperitoneal injection of streptozotocin (STZ), full-thickness excision wounds were made on the shaved dorsal skin. Different concentrations of WIN-1001X cream were topically applied (n=6/group). 3% WIN-1001X cream was more effective in promoting wound healing compared to the 1% and 5% formulations.


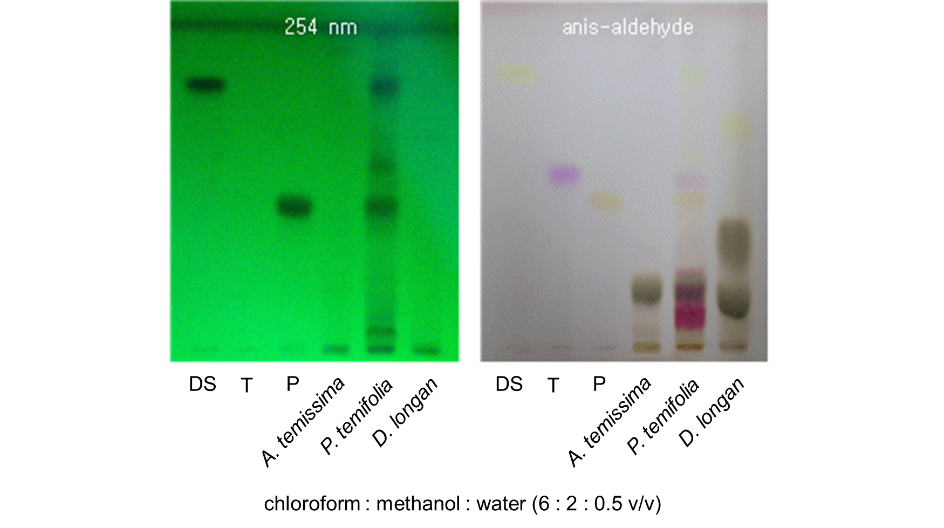
**Supplementary Figure 2**

**Supplementary Figure 2. Identification of *Polygala tenuifolia*.** To confirm the presence of *Polygala tenuifolia* in WIN-1001X, the concentrated extract sample was dissolved in 70% methanol to a final concentration of 100 mg/mL. The solution was spotted onto a Silica gel 60 F254 plate and developed using a solvent system of chloroform : methanol : water (6:3:0.5, v/v/v). The plate was examined under UV light at 254 nm and subsequently visualized by spraying with anisaldehyde-sulfuric acid reagent. Under short-wave UV light (254 nm), spots corresponding to 3',6-disinapoyl sucrose (DS) and polygalaxanthone III (P) were observed at Rf values of approximately 0.8 and 0.5, respectively. Upon anisaldehyde staining, a characteristic violet spot corresponding to tenuifolin (T) appeared at an Rf value of approximately 0.6. These compounds are known characteristic markers of *Polygala tenuifolia* listed in the pharmacopoeia. Furthermore, distinct violet spots observed near Rf 0.2 were specific to *Polygala tenuifolia* and were not present in *Angelica tenuissima* or *Dimocarpus longan*. Therefore, this method was applied as a specific identification test for *Polygala tenuifolia* within the WIN-1001X raw material.


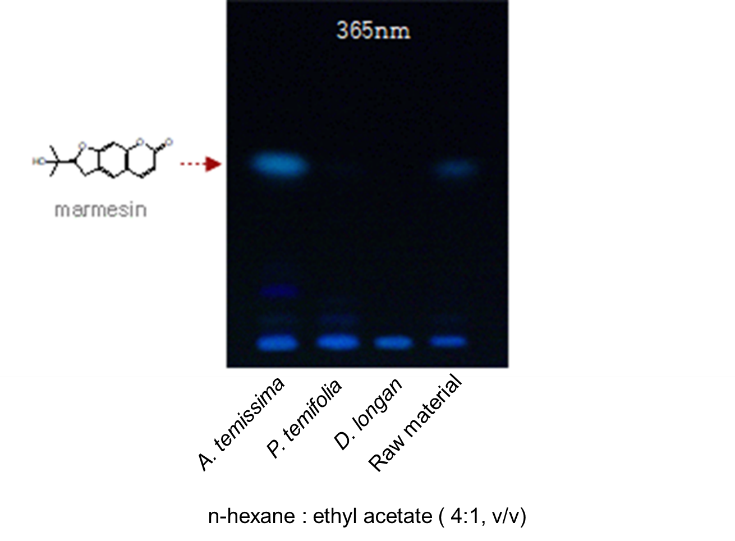
**Supplementary Figure 3**

**Supplementary Figure 3.** **Identification of *Angelica tenuissima.*** To confirm the presence of *Angelica tenuissima* in WIN-1001X, a sample was dissolved in 70% methanol to a concentration of 100 mg/mL. The solution was spotted onto a Silica gel 60 F254 plate and developed using a solvent system of n-hexane : ethyl acetate (4:1, v/v). The plate was visualized under UV light at 365 nm. A characteristic blue fluorescent spot corresponding to marmesin was observed at an Rf value of approximately 0.5. This spot is specific to *Angelica tenuissima* and was not detected in *Polygala tenuifolia* or *Dimocarpus longan*, thus confirming the specificity of this method for identifying *Angelica tenuissima* within the WIN-1001X extract.

**
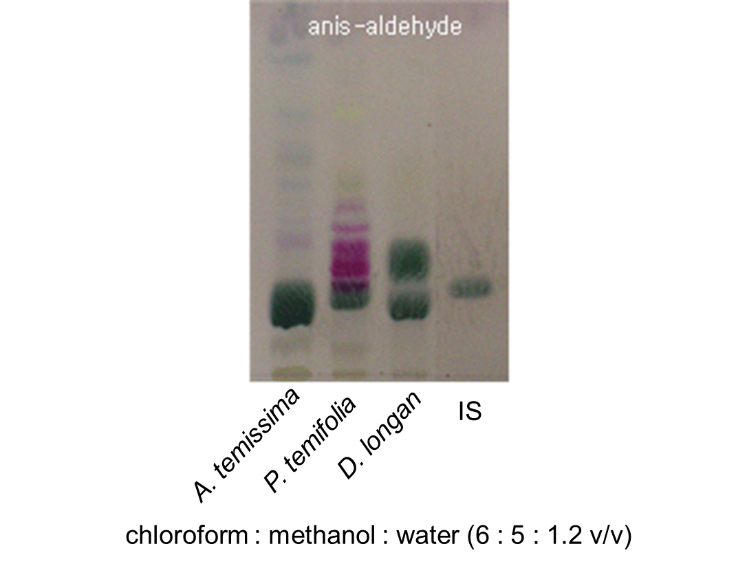
Supplementary Figure 4**

**Supplementary Figure 4. Identification of *Dimocarpus longan.*** To confirm the presence of *Dimocarpus longan* in WIN-1001X, the concentrated extract sample was dissolved in 70% methanol to a final concentration of 200 mg/mL. Sucrose was used as an internal standard (IS) and prepared at a concentration of 0.5 mg/mL. Both solutions were spotted onto a Silica gel 60 F254 plate. The plate was developed using a solvent system of chloroform : methanol : water (6:5:1.2, v/v/v) and subsequently visualized by spraying with anisaldehyde-sulfuric acid reagent. Upon visualization with anisaldehyde, dark green spots were observed in the *Dimocarpus longan* sample, appearing both above and below the position corresponding to the internal standard. Notably, the spot located above the internal standard was specific to *Dimocarpus longan* and was not detected in *Angelica tenuissima* or *Polygala tenuifolia*, thus confirming the specificity of this method for identifying *Dimocarpus longan* within the WIN-1001X extract

**
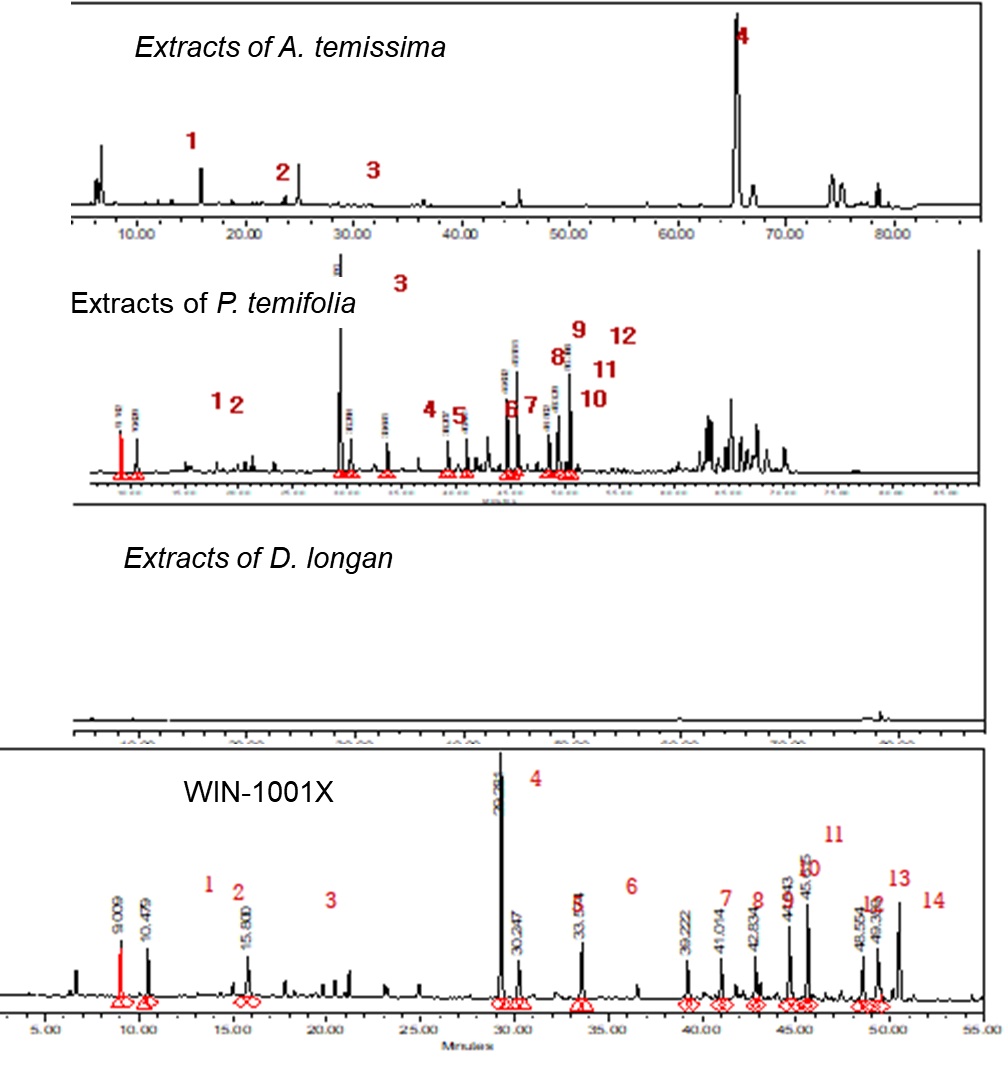
Supplementary Figure 5**

**Supplementary Figure 5. UPLC-based component profiling of WIN-1001X*.*** A component‑profiling method was developed for quality control of the herbal materials and raw ingredients. Major peaks (4 for *Angelica dahurica*, 12 for *Polygala tenuifolia*, and 14 for raw materials) were monitored for similarity analysis. According to the guidelines of the Korean Ministry of Food and Drug Safety, the acceptance criterion was set at a similarity value (r) ≥ 0.90.

**
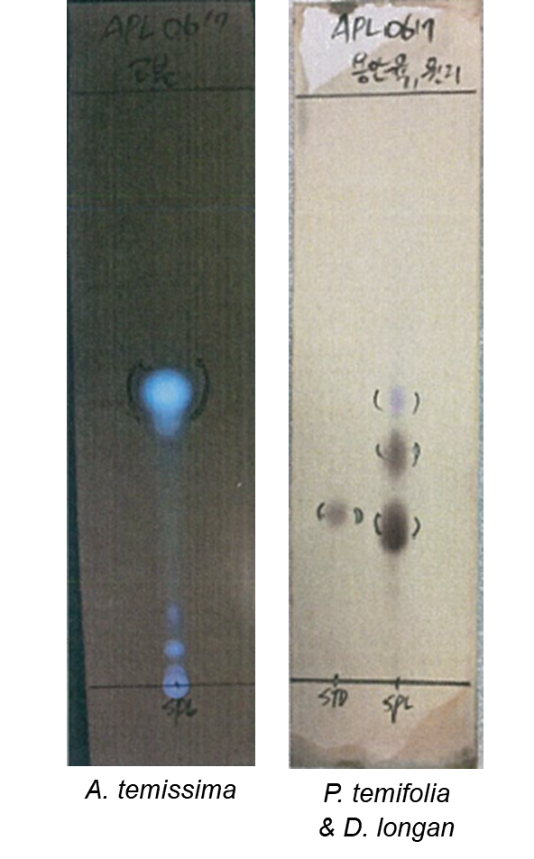
Supplementary Figure 6**

**Supplementary Figure 6. TLC-based identification of *Angelica dahurica*, *Polygala tenuifolia*, and *Dimocarpus longan.*** Identification of *Angelica dahurica*: Ten microliters of the test solution were applied to a silica gel TLC plate (Kieselgel 60 F254) and developed with hexane–ethyl acetate (4:1, v/v). Under UV light at 365 nm, a blue fluorescent spot appeared at an Rf value of approximately 0.5. Identification of *Polygala tenuifolia* and *Longan aril*: Ten microliters each of the test solution and internal standard were applied to a silica gel TLC plate (Kieselgel 60 F254) and developed with chloroform–methanol–water (6:5:1.2, v/v). After drying, the plate was sprayed with p‑anisaldehyde–sulfuric acid reagent and heated at 120 °C for color development. The internal standard appeared near Rf 0.25, the Longan aril showed a brown spot near Rf 0.3, and a purple spot above it corresponded to *Polygala tenuifolia*.

**Supplementary Figure 7**

**
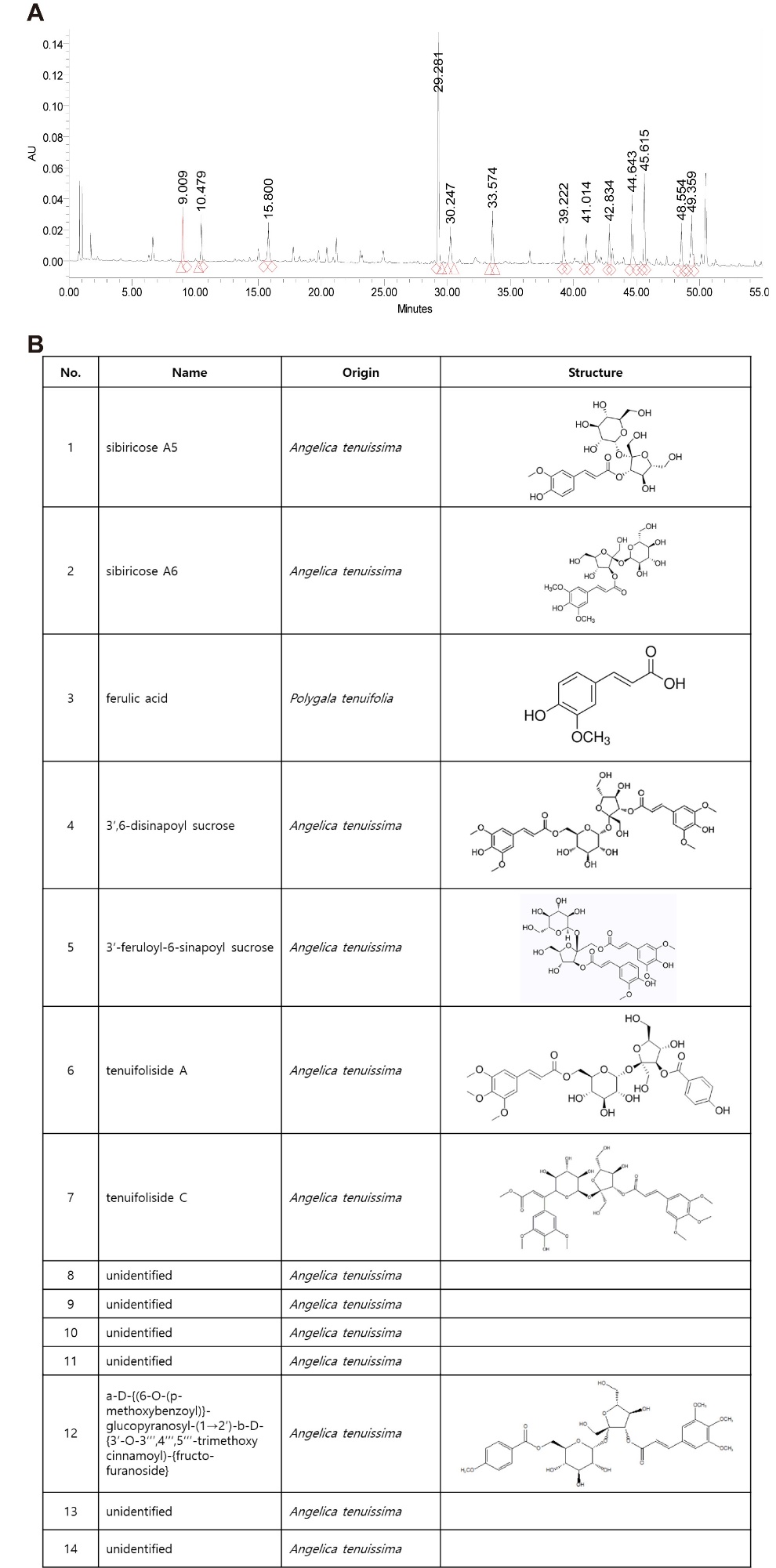
**

**Supplementary Figure 7. (A) UPLC-LS chromatogram of WIN-1001X. Total 14 major components were identified. (B) List of major components.**

**Supplemental Table 1**

Table S1. Oligonucleotide primers used for quantitative PCR in this study

| Gene | Forward | Reverse |
| --- | --- | --- |
| *IL-1β* | TGTGTAATGAAAGACGGCACA | GGGGAACTCTGCAGACTCAA |
| *IL-6* | TAGTCCTTCCTACCCCAATTTCC | TTGGTCCTTAGCCACTCCTTC |
| *IL-8* | CAAGGCTGGTCCATGCTCC | TGCTATCACTTCCTTTCTGTTGC |
| *IL-23α* | ATAGCCCCATGGAGCAACTT | TAGAACTCAGGCTGGGCATC |
| *TNFα* | GATTATGGCTCAGGGTCCAACT | GGACATTCGAGGCTCCAGTGA |
| *iNOS* | TCCTGGACATTACGACCCCT | AGGCCTCCAATCTCTGCCTA |
| *Arginase1* | AGAGACCACGGGGACCTGGC | TGGACCTCTGCCACCACACC |
| *Fizz1* | GGGATGACTGCTACTGGGTG | TCAACGAGTAAGCACAGGCA |
| *Defb1* | ACATGGAGGATTCTGTCTCCG | TGGGCTTATCTGGTTTACAGGTT |
| *Defb2* | TGTCAGAGCCATTTGTCCTCC | GCAACAGGGGTTCTTCTCTG |
| *Defb3* | TTGTTTGAGGAAAGGAGGCAGA | GGAACTCCACAACTGCCAATC |
| *LL-37* | CACAGCCCTTTCGGTTCAAG | TTCACCAATCTTCTCCCCACC |
| *PDGFβ* | GAGGAAGCCGAGATACCCC | TGCTGTGGATCTGACTTCGA |
| *HGF* | ATGTGGGGGACCAAACTTCTG | GGATGGCGACATGAAGCAG |
| *KGF* | CATGCTTCCACCTCGTCTGT | CAGTTCACACTCGTAGCCGT |
| *TGFβ* | CCACCTGCAAGACCATCGAC | CTGGCGAGCCTTAGTTTGGAC |
| *VEGFα* | GCACATAGGAGAGATGAGCTTCC | CTCCGCTCTGAACAAGGCT |
| *VEGFβ* | GCCAGACAGGGTTGCCATAC | GGAGTGGGATGGATGATGTCAG |
| *MMP1* | GCTCATGCTTTTCTGCCAGG | TAGAATGGGAGAGTCCAAGGG |
| *MMP2* | GATAACCTGGATGCCGTCGTG | CTTCACGCTCTTGAGACTTTG GTTC |
| *MMP3* | TGGAGATGCTCACTTTGACG | GCCTTGGCTGAGTGGTAGAG |
| *MMP8* | CACACTCCGTGGGGAGATTT | CCTGAAGACCGTTGGGTAGG |
| *MMP9* | TGTCTGGAGATTCGACTTGAAGTC | TGAGTTCCAGGGCACACCA |
| *MMP13* | TGTTTGCAGAGCACTACTTGA | CAGTCACCTCTAAGCCAAAGAA |
| *Filaggrin* | ATGTCCGCTCTCCTGGAAAG | TGGATTCTTCAAGACTGCCTGTA |
| *Loricrin* | CTCCTGTGGGTTGTGGAAAGA | TGGAACCACCTCCATAGGAAC |
| *Involucrin* | CAGCAGGAACAAGCAAAGCA | GGCCGCTTCTCCTCATGTTT |
| *Keratin1* | GACAAGGTGCGCTTCCTAGA | AAGTTTTGGGTCCGGGTTGT |
| *18s rRNA* | AGTCCCTGCCCTTTGTACACA | CGATCCGAGGGCCTCACTA |

**Supplemental Table 2**

Table S2. P‑values for All Figure‑Related Analyses

| Figure |  | Dunnett's multiple comparisions test | Summary | p value |
| --- | --- | --- | --- | --- |
| Figure 1C | D0 | control placebo vs. STZ placebo | ns | >0.9999 |
|  |  | STZ placebo vs. STZ WNX | ns | >0.9999 |
|  | D2 | control placebo vs. STZ placebo | ** | 0.0032 |
|  |  | STZ placebo vs. STZ WNX | * | 0.018 |
|  | D4 | control placebo vs. STZ placebo | * | 0.0228 |
|  |  | STZ placebo vs. STZ WNX | * | 0.0187 |
|  | D7 | control placebo vs. STZ placebo | * | 0.0261 |
|  |  | STZ placebo vs. STZ WNX | ns | 0.1015 |
|  | D9 | control placebo vs. STZ placebo | * | 0.0483 |
|  |  | STZ placebo vs. STZ WNX | ns | 0.2369 |
|  | D12 | control placebo vs. STZ placebo | ns | 0.0541 |
|  |  | STZ placebo vs. STZ WNX | ns | 0.0594 |
| Figure 2A | MPO | control placebo vs. STZ placebo | ** | 0.0014 |
|  |  | STZ placebo vs. STZ WNX | *** | 0.0005 |
| Figure 2B | CD68 | control placebo vs. STZ placebo | * | 0.0107 |
|  |  | STZ placebo vs. STZ WNX | ** | 0.0042 |
| Figure 2C | *IL-1β* | control placebo vs. STZ placebo | ** | 0.0059 |
|  |  | STZ placebo vs. STZ WNX | * | 0.0223 |
|  | *IL-6* | control placebo vs. STZ placebo | *** | <0.0001 |
|  |  | STZ placebo vs. STZ WNX | *** | <0.0001 |
|  | *IL-8* | control placebo vs. STZ placebo | *** | <0.0001 |
|  |  | STZ placebo vs. STZ WNX | *** | <0.0001 |
|  | *IL-23* | control placebo vs. STZ placebo | *** | <0.0001 |
|  |  | STZ placebo vs. STZ WNX | *** | <0.0001 |
|  | *TNFα* | control placebo vs. STZ placebo | *** | <0.0001 |
|  |  | STZ placebo vs. STZ WNX | *** | <0.0001 |
|  | *iNOS* | control placebo vs. STZ placebo | *** | <0.0001 |
|  |  | STZ placebo vs. STZ WNX | *** | <0.0001 |
| Figure 3 2 Days | *Defb1* | control placebo vs. STZ placebo | ns | 0.04638 |
|  |  | STZ placebo vs. STZ WNX | ns | 0.9608 |
|  | *Defb2* | control placebo vs. STZ placebo | ns | 0.2249 |
|  |  | STZ placebo vs. STZ WNX | ns | 0.8811 |
|  | *Defb3* | control placebo vs. STZ placebo | *** | <0.0001 |
|  |  | STZ placebo vs. STZ WNX | ns | 0.9415 |
|  | *LL-37* | control placebo vs. STZ placebo | ns | 0.6496 |
|  |  | STZ placebo vs. STZ WNX | ns | 0.2787 |
| Figure 3  7 days | *Defb1* | control placebo vs. STZ placebo | ns | 0.4174 |
|  |  | STZ placebo vs. STZ WNX | *** | <0.0001 |
|  | *Defb2* | control placebo vs. STZ placebo | ns | 0.6262 |
|  |  | STZ placebo vs. STZ WNX | ** | 0.0026 |
|  | *Defb3* | control placebo vs. STZ placebo | ns | 0.2806 |
|  |  | STZ placebo vs. STZ WNX | *** | 0.0004 |
|  | *LL-37* | control placebo vs. STZ placebo | *** | 0.0009 |
|  |  | STZ placebo vs. STZ WNX | * | 0.0343 |
| Figure 4A | iNOS | control placebo vs. STZ placebo | *** | <0.0001 |
|  |  | STZ placebo vs. STZ WNX | *** | <0.0001 |
| Figure 4B | Arg 1 | control placebo vs. STZ placebo | *** | <0.0001 |
|  |  | STZ placebo vs. STZ WNX | *** | <0.0001 |
| Figure 4C | *Arginase 1* | control placebo vs. STZ placebo | ns | 0.7545 |
|  |  | STZ placebo vs. STZ WNX | *** | 0.0001 |
|  | *Fizz 1* | control placebo vs. STZ placebo | ** | 0.001 |
|  |  | STZ placebo vs. STZ WNX | *** | <0.0001 |
| Figure 5A | VEGF | control placebo vs. STZ placebo | ** | 0.0012 |
|  |  | STZ placebo vs. STZ WNX | *** | <0.0001 |
| Figure 5B | *VEGFα* | control placebo vs. STZ placebo | ns | 0.4609 |
|  |  | STZ placebo vs. STZ WNX | *** | <0.0001 |
|  | *VEGFβ* | control placebo vs. STZ placebo | * | 0.0191 |
|  |  | STZ placebo vs. STZ WNX | ** | 0.0023 |
| Figure 5C | PCNA | control placebo vs. STZ placebo | *** | <0.0001 |
|  |  | STZ placebo vs. STZ WNX | *** | <0.0001 |
| Figure 5D | *PDGFβ* | control placebo vs. STZ placebo | ns | 0.9522 |
|  |  | STZ placebo vs. STZ WNX | ** | 0.001 |
|  | *HGF* | control placebo vs. STZ placebo | ns | 0.9185 |
|  |  | STZ placebo vs. STZ WNX | *** | 0.0003 |
|  | *KGF* | control placebo vs. STZ placebo | *** | 0.0005 |
|  |  | STZ placebo vs. STZ WNX | *** | <0.0001 |
|  | *TGF-β* | control placebo vs. STZ placebo | ** | 0.0062 |
|  |  | STZ placebo vs. STZ WNX | *** | <0.0001 |
| Figure 5E | H&E | control placebo vs. STZ placebo | ** | 0.001 |
|  |  | STZ placebo vs. STZ WNX | ** | 0.0088 |
| Figure 5F | α-SMA | control placebo vs. STZ placebo | *** | <0.0001 |
|  |  | STZ placebo vs. STZ WNX | *** | <0.0001 |
| Figure 6A | K17 | control placebo vs. STZ placebo | * | 0.0296 |
|  |  | STZ placebo vs. STZ WNX | ** | 0.0025 |
| Figure 6B | *Filaggrin* | control placebo vs. STZ placebo | ns | 0.8634 |
|  |  | STZ placebo vs. STZ WNX | *** | <0.0001 |
|  | *Loricrin* | control placebo vs. STZ placebo | ns | 0.7501 |
|  |  | STZ placebo vs. STZ WNX | *** | <0.0001 |
|  | *Involucrin* | control placebo vs. STZ placebo | ns | 0.475 |
|  |  | STZ placebo vs. STZ WNX | *** | <0.0001 |
|  | *Keratin 1* | control placebo vs. STZ placebo | ns | 0.8548 |
|  |  | STZ placebo vs. STZ WNX | * | 0.0228 |
| Sup. 1B | D0 | control placebo vs. STZ placebo | ns | >0.9999 |
|  |  | STZ placebo vs. 1% WNX cream | ns | >0.9999 |
|  |  | STZ placebo vs. 3% WNX cream | ns | >0.9999 |
|  |  | STZ placebo vs. 5% WNX cream | ns | >0.9999 |
|  | D2 | control placebo vs. STZ placebo | *** | <0.0001 |
|  |  | STZ placebo vs. 1% WNX cream | ns | 0.6446 |
|  |  | STZ placebo vs. 3% WNX cream | *** | <0.0001 |
|  |  | STZ placebo vs. 5% WNX cream | ns | 0.6003 |
|  | D4 | control placebo vs. STZ placebo | *** | <0.0001 |
|  |  | STZ placebo vs. 1% WNX cream | ns | 0.7078 |
|  |  | STZ placebo vs. 3% WNX cream | *** | <0.0001 |
|  |  | STZ placebo vs. 5% WNX cream | ns | 0.1158 |
|  | D7 | control placebo vs. STZ placebo | * | 0.0302 |
|  |  | STZ placebo vs. 1% WNX cream | ns | 0.5196 |
|  |  | STZ placebo vs. 3% WNX cream | * | 0.0345 |
|  |  | STZ placebo vs. 5% WNX cream | ns | 0.4445 |
|  | D9 | control placebo vs. STZ placebo | ns | 0.1149 |
|  |  | STZ placebo vs. 1% WNX cream | ns | 0.5022 |
|  |  | STZ placebo vs. 3% WNX cream | ns | 0.1871 |
|  |  | STZ placebo vs. 5% WNX cream | ns | 0.5798 |
|  | D12 | control placebo vs. STZ placebo | ns | 0.8667 |
|  |  | STZ placebo vs. 1% WNX cream | ns | 0.9488 |
|  |  | STZ placebo vs. 3% WNX cream | ns | 0.8692 |
|  |  | STZ placebo vs. 5% WNX cream | ns | 0.9554 |

**Supplemental Table 3**

Table S3. WIN-1001X Toxicity Assessment under GLP Conditions (Oral and Topical)


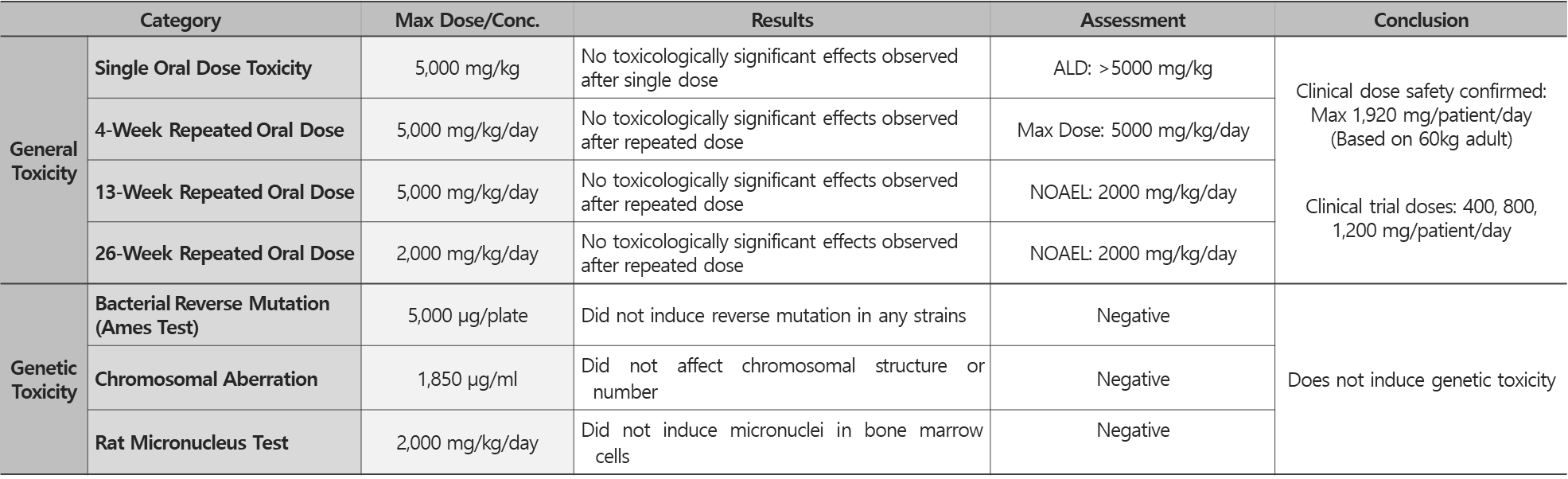


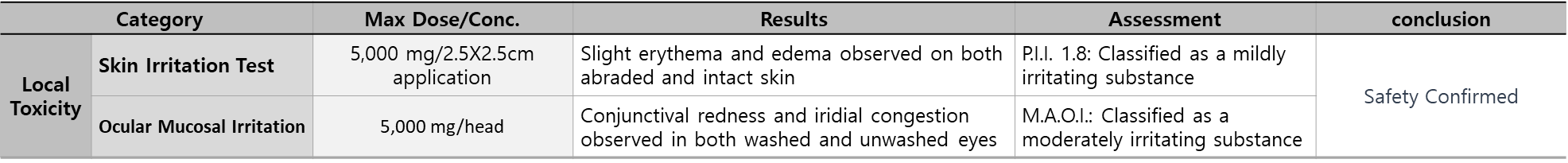

Supplement: Supplementary file 1 [file Supplementaryfile1.docx]
